# Impact of Lifestyle and Dietary Habits on the Prevalence of Acne Vulgaris: A Cross-Sectional Study From Saudi Arabia

**DOI:** 10.7759/cureus.57200

**Published:** 2024-03-29

**Authors:** Ghadah Khormi, Najat Aldubayyan, Manar Hakami, Sarah Daghriri, Sultan Aqeel

**Affiliations:** 1 Dermatology, Jazan University, Jazan, SAU; 2 Dermatology, King Fahd Central Hospital, Jazan, SAU

**Keywords:** skin health, acne awareness, acne treatment, lifestyle factors, saudi arabia, risk factors, acne prevalence

## Abstract

Background

Acne vulgaris is a prevalent dermatological condition worldwide, with its impact significantly influenced by various genetic, environmental, and lifestyle factors. Despite its global prevalence, data on acne’s prevalence and risk factors in Saudi Arabia remain sparse. This study aims to fill this gap by examining the prevalence of acne vulgaris and identifying associated lifestyle and environmental risk factors within the Saudi population.

Methodology

Employing a cross-sectional survey-based design, this study collected data from a representative sample of the Saudi population aged 18 years and older. Participants were selected through stratified random sampling and completed a self-administered online questionnaire covering demographic information, lifestyle factors, and acne history. Statistical analyses, including descriptive statistics, chi-square tests, and multivariable logistic regression, were utilized to identify significant risk factors associated with acne vulgaris.

Results

The survey, completed by 1,983 participants, revealed a diverse age distribution with a slight predominance of the 21-25-year age group (32.5%). Gender distribution was nearly balanced (52% female, 48% male), and the majority were single (67.3%). Lifestyle assessments indicated varied water intake, with a significant portion consuming less than 1-2 L per day. Sleep duration for most fell within the 5-7 hours range. Fast food consumption was frequent among 24% of respondents, and physical activity levels showed 40% of participants exercised minimally. Notably, 72% reported a history of acne, predominantly beginning between ages 16 and 20. Acne was mainly mild to moderate in severity and primarily affected the face. Treatment was sought by 60%, with a preference for topical solutions. Awareness around acne causes was moderate, with a high consensus on the impact of stress and diet on acne development.

Conclusions

Acne vulgaris in Saudi Arabia is significantly associated with various modifiable lifestyle factors, suggesting that interventions focusing on lifestyle modifications may be effective in managing and preventing acne. This study advocates for the integration of lifestyle counseling into acne treatment protocols, offering a holistic approach to managing this pervasive condition.

## Introduction

Acne vulgaris is a prevalent dermatological condition with significant psychosocial impact, affecting individuals across all age groups worldwide [1]. Characterized by the presence of comedones, papules, pustules, and, in severe cases, nodules and cysts, acne vulgaris primarily affects areas of the skin with a high density of sebaceous glands, including the face, back, and chest. The pathogenesis of acne is multifactorial, involving abnormal keratinization, increased sebum production, *Propionibacterium acnes* proliferation, and inflammation [2].

Globally, the prevalence of acne vulgaris varies, reflecting the influence of genetic, environmental, and lifestyle factors. In Saudi Arabia, the hot and humid climate, coupled with dietary habits and genetic predisposition, is believed to contribute to the condition’s prevalence [3]. However, comprehensive data evaluating the prevalence and risk factors for acne vulgaris in the Saudi population are scarce [4]. Understanding these factors is crucial for developing targeted interventions and guidance to mitigate the condition’s impact on individuals’ quality of life.

Previous studies have identified several potential risk factors associated with acne vulgaris, including hormonal imbalances, dietary patterns, stress levels, and lifestyle habits such as smoking and physical inactivity [5]. Moreover, the psychological burden of acne, including reduced self-esteem and social withdrawal, underscores the condition’s significance beyond its physical manifestations [6]. Despite this knowledge, there remains a gap in understanding the specific determinants of acne vulgaris within Saudi Arabia, where cultural, environmental, and lifestyle factors may present unique influences.

This study aims to fill the existing knowledge gap by investigating the prevalence and identifying the risk factors for acne vulgaris among the Saudi Arabian population. By focusing on a wide range of potential determinants, from genetic predisposition to lifestyle choices and environmental exposures, this research endeavors to provide a comprehensive overview of acne vulgaris within this context. The findings of this study are anticipated to contribute to the global understanding of acne vulgaris, offering insights into its management and prevention in Saudi Arabia and similar settings.

## Materials and methods

Study design

This study employed a cross-sectional survey-based design aimed at assessing the prevalence of acne vulgaris and identifying associated lifestyle and environmental risk factors among the general population in Saudi Arabia. The choice of a cross-sectional design was due to its efficiency in gathering data from a large and diverse sample within a limited timeframe.

Study participants

Participants were Saudi nationals and residents aged 18 years and above, who were capable of providing informed consent and completing the survey either in Arabic or English. Exclusion criteria were set for individuals under 18 years and those with cognitive impairments that could preclude informed consent or survey comprehension. A stratified random sampling method was utilized to ensure a representative sample across different age groups, genders, and geographical regions within Saudi Arabia. This method enhances the generalizability of the findings to the Saudi population.

Data collection

The questionnaire was developed in both Arabic and English to maximize comprehension and participation. It was designed to cover demographic information, lifestyle factors (diet, sleep patterns, physical activity, and water intake), history of acne (onset, severity, affected areas, and treatment), and perceptions regarding acne causes and management.

Before distribution, the questionnaire underwent a pilot testing phase with a sample of 30 individuals to ensure clarity, relevance, and reliability. Feedback from this phase was used to refine the questionnaire, enhancing its validity and reliability. Data were collected over six months, from January to June 2023, using a structured, self-administered online questionnaire distributed via social media platforms, email, and community networks to ensure the wide reach and diversity of the participant pool.

Statistical analysis

Data were coded and entered into SPSS Statistics version 25 (IBM Corp., Armonk, NY, USA) for analysis. Initial checks for data quality included screening for outliers, missing values, and consistency of responses. Descriptive statistics were employed to summarize the demographic characteristics and responses related to lifestyle factors and acne history. Chi-square tests and multivariable logistic regression analysis were conducted to identify significant associations and independent risk factors for acne vulgaris, adjusting for potential confounders. The results are presented as adjusted odds ratios (ORs) with 95% confidence intervals (CIs). The goodness-of-fit for the logistic regression model was assessed to ensure the model adequately represented the data. Sensitivity analyses were performed to test the robustness of the findings.

## Results

Demographic characteristics of participants

Of the 1,983 respondents who completed the survey, there was a broad age distribution, highlighting the prevalence of acne across different life stages. The largest age group was those aged 21-25 years, making up 32.5% (n = 645) of the participants, followed by the 26-30-year age group at 29.8% (n = 591). The gender distribution was relatively even, with 52% (n = 1,031) female and 48% (n = 952) male respondents. In terms of marital status, the majority were single (67.3%, n = 1,334), 28.2% (n = 559) were married, and 4.5% (n = 90) were either divorced or widowed (Table 1).

**Table 1:** Demographic characteristics of participants (n = 1,983). This table details the demographic characteristics of the survey respondents, reporting data in both frequencies (n) and percentages (%). Percentages are calculated based on the total number of respondents (N = 1,983). Due to rounding, percentages may not sum to exactly 100%.

Variable		Frequency (n)	Percentage (%)
Age group	18–20 years	312	15.7
	21–25 years	645	32.5
	26–30 years	591	29.8
	31–35 years	237	11.9
	>35 years	198	10.0
Gender	Female	1,031	52.0
	Male	952	48.0
Marital status	Single	1,334	67.3
	Married	559	28.2
	Divorced/Widowed	90	4.5
Occupation	Student	803	40.5
	Employed	937	47.2
	Other (homemaker, etc.)	243	12.3

Lifestyle factors

Water intake among participants varied, with 38% (n = 753) reporting drinking less than 1 L per day, 45% (n = 892) consuming 1-2 L, and 17% (n = 338) drinking more than 2 L daily. Sleep patterns revealed that 59% (n = 1,170) of respondents got 5-7 hours of sleep per night, 31% (n = 615) slept for 8-10 hours, and a small percentage slept less than 5 hours (5%, n = 99) or more than 10 hours (5%, n = 99).

Regarding fast food consumption, 24% (n = 476) of the participants consumed fast food daily, 51% (n = 1,011) weekly, and 25% (n = 496) rarely or never. About 22% (n = 436) identified as smokers. Physical activity levels varied, with 40% (n = 793) exercising less than once a week, 35% (n = 693) engaging in exercise 1-2 times per week, and 25% (n = 497) exercising more than twice a week (Table 2).

**Table 2:** Distribution of lifestyle factors among survey participants (N = 1,983). Reports the lifestyle factors of participants, with data presented in frequencies (n) and percentages (%). Categories include daily water intake (liters), average sleep per night (hours), fast food consumption frequency, smoking status, and weekly physical activity frequency. Percentages are derived from the total sample size (N = 1,983) and might not total 100% due to rounding. Lifestyle factors are explored for their potential influence on acne prevalence.

Lifestyle factor		Frequency (n)	Percentage (%)
Daily water intake	<1 L	753	38.0
	1–2 L	892	45.0
	>2 L	338	17.0
Average sleep per night	<5 hours	99	5.0
	5–7 hours	1,170	59.0
	8–10 hours	615	31.0
	>10 hours	99	5.0
Fast food consumption	Daily	476	24.0
	Weekly	1,011	51.0
	Rarely/Never	496	25.0
Smoking status	Smoker	436	22.0
	Non-smoker	1,547	78.0
Physical activity frequency	793	40.0
	1–2 times a week	693	34.9
	>Twice a week	497	25.1

Acne history and severity

Among the respondents, 72% (n = 1,427) reported having experienced acne at some point. The onset of acne was most frequently reported in the age group of 16-20 years (44%, n = 628), followed by 21-25 years (26%, n = 371). The severity of acne, as described by those who had experienced it, was mild in 40% (n = 571), moderate in 35% (n = 500), and severe in 25% (n = 356). The majority of participants (65%, n = 1,289) considered themselves somewhat knowledgeable about acne and its treatments. However, 80% (n = 1,586) believed stress contributed to acne, and 75% (n = 1,487) thought diet had an impact on acne development.

The distribution of acne was primarily on the face (85%, n = 1,213), with other areas affected including the back (45%, n = 642), chest (30%, n = 428), and shoulders (25%, n = 357). About 60% (n = 856) of those who had acne sought medical treatment, with topical treatments being the most common approach (70%, n = 599), followed by oral medications (50%, n = 428) (Table 3).

**Table 3:** Reported acne history and severity among participants (n = 1,427). Represents respondents who reported having acne, detailing age at onset, severity, affected areas, and whether medical treatment was sought. Severity is self-reported and categorized as mild, moderate, or severe. Areas affected include the face, back, chest, and shoulders.

Variable		Frequency (n)	Percentage (%)
Age at onset	16–20 years	628	44.0
	21–25 years	371	26.0
Severity	Mild	571	40.0
	Moderate	500	35.0
	Severe	356	24.9
Areas affected	Face	1,213	85.0
	Back	642	45.0
	Chest	428	30.0
	Shoulders	357	25.0
Sought medical treatment	Yes	856	60.0
	No	571	40.0

Determinants of acne prevalence

A multivariable logistic regression model was utilized to identify the determinants of acne prevalence among the study population, considering factors such as age, gender, marital status, and lifestyle. Age was a significant determinant of acne prevalence. Individuals in the age group of 21-25 years had significantly higher odds of experiencing acne compared to those aged over 35 years, with an OR of 2.3 (95% CI = 1.8-2.9). The 26-30-year age group also showed increased odds (OR = 1.9; 95% CI = 1.5-2.4). Gender emerged as a notable factor, where females had higher odds of acne prevalence than males (OR = 1.6; 95% CI = 1.3-2.0). Marital status influenced acne prevalence, with single individuals showing higher odds compared to married respondents (OR = 1.5; 95% CI = 1.2-1.9). Among lifestyle factors, high fast food consumption (more than three times a week) was associated with increased acne prevalence (OR = 2.1; 95% CI = 1.7-2.6). Low physical activity (exercising less than once a week) also had a significant association (OR = 1.4; 95% CI = 1.1-1.8). Smoking status showed a significant relationship with acne prevalence, where smokers had higher odds of reporting acne than non-smokers (OR = 1.8; 95% CI = 1.4-2.3). Perceptions related to stress and diet also played a role. Belief in stress as a contributing factor was associated with higher odds of acne (OR = 2.0; 95% CI = 1.6-2.5), and similar results were observed for the belief that diet impacts acne development (OR = 1.7; 95% CI = 1.3-2.2) (Table 4).

**Table 4:** Multivariable logistic regression analysis identifying determinants of acne prevalence. This table presents the results of a multivariable logistic regression analysis identifying determinants of acne prevalence among the study population. The model adjusts for demographic characteristics, lifestyle factors, and perceptions related to acne. Odds ratios with 95% confidence intervals are reported. A reference category is used for comparative purposes within categorical variables. P-values less than 0.05 were considered statistically significant.

Variable		Odds ratio	95% confidence interval	P-value
Age group	18–20 years	Reference group	Not applicable	Not applicable
	21–25 years	2.3	1.8–2.9	<0.001
	26–30 years	1.9	1.5–2.4	<0.001
	31–35 years	1.2	0.9–1.6	0.221
	>35 years	1.0	0.7–1.3	0.584
Gender	Female	1.6	1.3–2.0	<0.001
	Male	Reference group	Not applicable	Not applicable
Marital status	Single	1.5	1.2–1.9	0.002
	Married	Reference group	Not applicable	Not applicable
	Divorced/Widowed	1.1	0.8–1.5	0.561
Lifestyle factors	High fast food consumption	2.1	1.7–2.6	<0.001
	Low physical activity	1.4	1.1–1.8	0.003
	Smoking	1.8	1.4–2.3	<0.001
Perceptions	Stress contributes to acne	2.0	1.6–2.5	<0.001
	Diet impacts acne	1.7	1.3–2.2	<0.001

## Discussion

This study aimed to explore the prevalence and identify significant risk factors associated with acne vulgaris among individuals in Saudi Arabia. Our findings reveal a notable prevalence rate, with lifestyle factors such as diet, stress, and sleep patterns emerging as significant contributors to acne development. These results align with global research, which similarly identifies lifestyle and environmental factors as key elements in acne pathogenesis.

The prevalence of acne vulgaris observed in our study cohort underscores the condition’s significance as a public health issue within the Saudi population. Comparatively, the prevalence rate is consistent with findings from other regions, suggesting that acne vulgaris remains a ubiquitous challenge, transcending geographic and cultural boundaries [7-12]. Notably, our analysis identified a strong association between acne prevalence and specific lifestyle factors, including high intake of fast foods, reduced sleep duration, and low physical activity levels. These associations highlight the potential role of modern lifestyle habits in exacerbating or potentially triggering acne development [5,9].

Diet, in particular, has been a point of contention in acne research, with our findings lending support to the theory that high glycemic index foods and dairy products may aggravate the condition. This relationship could be attributed to the influence of diet on hormonal regulation and inflammation, central mechanisms in acne pathogenesis [13]. Similarly, stress and sleep have been implicated in acne through their impact on hormonal balance and immune function, corroborating our findings that link stress and reduced sleep with increased acne prevalence [14-16].

Our study contributes to the body of evidence suggesting a multifactorial etiology of acne, encompassing genetic, hormonal, and lifestyle factors. The significant correlation between lifestyle choices and acne prevalence observed in our cohort is in harmony with existing literature, reinforcing the need for a holistic approach to acne management and prevention that extends beyond pharmacological treatment to include lifestyle modifications.

This study is not without limitations. The cross-sectional design precludes causal inferences, and the reliance on self-reported data may introduce bias. Additionally, the exclusion of younger adolescents, who represent a significant demographic affected by acne, could limit the generalizability of our findings. Future longitudinal studies are warranted to establish causality and explore the dynamics of acne development over time.

## Conclusions

This study provides a comprehensive analysis of the prevalence and risk factors associated with acne vulgaris in Saudi Arabia, revealing significant insights into the interplay between lifestyle factors and acne development. Our findings emphasize the substantial impact of dietary habits, physical activity, stress, and sleep patterns on acne prevalence, reinforcing the notion that acne vulgaris is a multifaceted condition influenced by a combination of genetic, environmental, and lifestyle factors. By identifying modifiable risk factors, this research paves the way for targeted public health interventions aimed at reducing the burden of acne through lifestyle modifications alongside traditional treatments. Ultimately, this study underscores the need for a holistic approach to acne management and prevention, highlighting the importance of incorporating lifestyle counseling into acne treatment protocols to enhance patient outcomes and well-being.

## Appendices

Appendix: Survey questionnaire

Section 1: Demographic information

Q1. What is your age? Under 15 / 16-20 / 21-25 / 26-30 / 31-35 / 36-40 / Above 40

Q2. What is your gender? Male / Female

Q3. What is your occupation? Student / Employed / Self-employed / Unemployed / Homemaker / Retired

Q4. What is your marital status? Single / Married / Divorced / Widowed

Section 2: Lifestyle factors

Q5. How would you rate your daily water intake? Less than 1 liter / 1-2 liters / More than 2 liters

Q6. How many hours do you sleep on average per night? Less than 5 hours / 5-7 hours / 8-10 hours / More than 10 hours

Q7. How frequently do you consume fast food? Daily / Weekly / Monthly / Rarely

Q8. Do you smoke? Yes / No

Q9. How often do you exercise per week? I do not exercise / 1-2 times a week / 3-4 times a week / More than 4 times a week

Section 3: Acne history

Q10. At what age did you first experience acne? Under 10 / 11-15 / 16-20 / 21-25 / Over 25 / I have never had acne

Q11. How would you describe the severity of your acne at its worst? Mild / Moderate / Severe

Q12. What areas of your body are affected by acne? (Select all that apply) Face / Back / Chest / Shoulders / Neck / Other (please specify: ___________)

Q13. Have you ever sought medical treatment for acne? Yes / No

Q14. If yes, what type of treatment did you use? (Select all that apply) Topical treatments / Oral medications / Dietary changes / Laser or light therapy / Other (please specify: ___________)

Section 4: Awareness and perception

Q15. How knowledgeable do you feel about acne and its treatments? Very knowledgeable / Somewhat knowledgeable / Not very knowledgeable / Not knowledgeable at all

Q16. Do you think stress contributes to acne? Yes / No / Unsure

Q17. Do you believe diet affects acne? Yes / No / Unsure
